# *Small and round seed 5 *gene encodes alpha-tubulin regulating seed cell elongation in rice

**DOI:** 10.1186/1939-8433-5-4

**Published:** 2012-02-27

**Authors:** Shuhei Segami, Izumi Kono, Tsuyu Ando, Masahiro Yano, Hidemi Kitano, Kotaro Miura, Yukimoto Iwasaki

**Affiliations:** 1 Faculty of Biotechnology, Fukui Prefectural University, 4-1-1 Kenjojima, Matsuoka, Eiheiji-Town, Fukui 910-1195, Japan; 2 Institute of the Society for Techno-innovation of Agriculture, Forestry and Fisheries, 446-1 Ippaizuka, Kamiyokoba, Tsukuba, Ibaraki 305-0854, Japan; 3 Laboratory of Synaptic Plasticity and Connectivity RIKEN Brain Science Institute, 2-1 Hirosawa, Wako, Saitama 351-0198, Japan; 4 National Institute of Agrobiological Science, 2-1-1 Kannondai, Tsukuba, Ibaraki 305-8602, Japan; 5 National Institute of Agrobiological Science, 1-2 Ohwashi, Tsukuba, Ibaraki 305-8634 Japan; 6 Bioscience and Biotechnology Center, Nagoya University, Furo, Chikusa, Nagoya, Aichi 464-8601, Japan

## Abstract

Seed size is an important trait in determinant of rice seed quality and yield. In this study, we report a novel semi-dominant mutant *Small and round seed 5 *(*Srs5*) that encodes alpha-tubulin protein. Lemma cell length was reduced in *Srs5 *compared with that of the wild-type. Mutants defective in the G-protein alpha subunit (*d1-1*) and brassinosteroid receptor, BRI1 (*d61-2*) also exhibited short seed phenotypes, the former due to impaired cell numbers and the latter due to impaired cell length. Seeds of the double mutant of *Srs5 *and *d61-2 *were smaller than those of *Srs5 *or *d61-2*. Furthermore, *SRS5 *and *BRI1 *genes were highly expressed in *Srs5 *and *d61-2 *mutants. These data indicate that *SRS5 *independently regulates cell elongation of the brassinosteroid signal transduction pathway

## Background

Seed size and weight are important traits for rice yield (Song and Ashikari 2008, Takeda and Matsuoka 2008). Several quantitative trait loci (QTLs) affecting seed size have been identified, namely *GW2 *encoding a RING-type protein that functions as an E3 ubiquitin ligase (Song *et al*. 2007), *qSW5 *encoding a novel protein with no known domains (Shoumura *et al*. 2008), and *GS3 *encoding a membrane protein with various conserved domains (Fan *et al*. 2006, Takano-Kai *et al*. 2009). Loss of *GW2 *and *qSW5 *function leads to a wider seed phenotype, and loss of *GS3 *function leads to a longer seed phenotype, both resulting in increased yield.

Causal genes of the small (or short) seed mutants have also been identified, namely *d1 *(also named *RGA1*) encoding the heterotrimeric G protein alpha subunit (Ashikari *et al*. 1999, Fujisawa *et al*. 1999), *d11 *encoding a cytochrome P450 involved in brassinosteroid (BR) biosynthesis (Tanabe *et al*. 2005), *d2 *and *brd2 *encoding another type of cytochrome P450 involved in BR synthesis (Hong et al. 2003, Hong *et al*. 2005), *d61 *(also named *OsBRI1*) encoding the BR receptor (Yamamuro *et al*. 2000), *srs1 *encoding a novel protein that has no known functional domains (Abe *et al*. 2010), and finally, *srs3 *encoding a kinesin 13 protein (Kitagawa *et al*. 2010). During seed formation in rice, it was demonstrated that *D1 *regulates cell number (Izawa *et al*. 2010), and *SRS1 *and *SRS3 *regulate cell length (Abe *et al*. 2010, Kitagawa *et al*. 2010). From these observations, *SRS1 *and *SRS3 *seem to affect seed size through signaling pathways other than G-protein signal transduction.

Although several genes regulating seed size have been identified, their molecular network underlying seed formation remains unclear. Here we report molecular cloning of a novel small and round seed mutant in *Srs5 *(*Small and round seed 5*). The results clearly demonstrated that *Srs5 *encodes alpha-tubulin and regulates cell elongation in rice seed.

## Results

### Characterization of the *Srs5 *mutant

**Figure 1:**
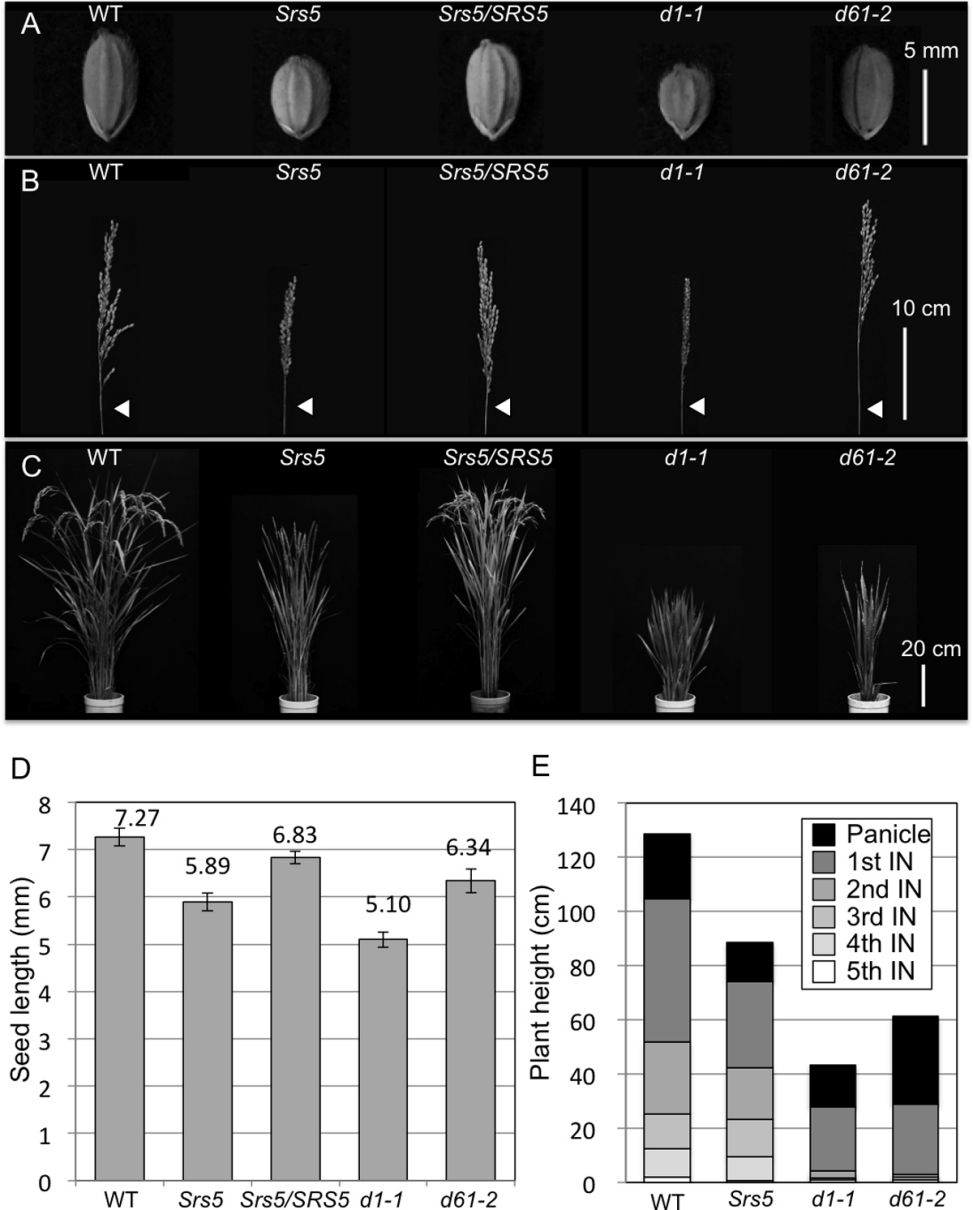
***Srs5 *mutant phenotypes**. (A) Seed morphology of T65, *Srs5*, *Srs5/SRS5*, *d1-1*, and *d61-2*. Bar = 5 mm. (B) Panicle morphology of T65, *Srs5, Srs5/SRS5, d1-1*, and *d61-2*. Arrowheads indicate panicle neck nodes. Bar = 10 cm. (C) Gross morphology of T65, *Srs5*, *Srs5/SRS5*, *d1-1*, and *d61-2*. Bar = 20 cm. (D) Seed length of T65, *Srs5*, *Srs5/SRS5*, *d1-1*, and *d61-2*. Numbers on graphs indicate average seed length ± S.D. (E) Internode length relative to the total length of the culm. Schematic representation of the internode elongation pattern of T65, *Srs5*, *d1-1*, and *d61-2*. IN: internode.

A mutant line, Kyudai No. 37, was identified by screening of small or short seed mutants from the rice collections of Togo Field, Nagoya University, and renamed *Small and round seed 5*, (*Srs5*). *Srs5 *shows shorter and rounder seeds, a shorter panicle and semi-dwarf plant phenotype, compared to WT (Figure 1A-C). Additionally, F_1 _plants derived from a cross between WT and *Srs5 *plants show intermediate seed length of parents seeds (Figure 1A and 1D). From these results, we presumed that the *Srs5 *mutation acts as semi-dominant gene. These phenotypes, short seed, short panicle, and dwarfism, are also exhibited by *d1-1 *and *d61-2 *mutants (Figure 1A-D). Comparison of internode elongation patterns among *Srs5, d1-1*, and *d61-2 *revealed that the internode elongation pattern of *Srs5 *differs from that of *d1-1 *and *d61-2 *(Figure 1E). Although *d1-1 *and *d61-2 *exhibit extremely stunted second, third, and fourth internodes, *Srs5 *shows equally shortened internodes (Figure 1E).

**Figure 2:**
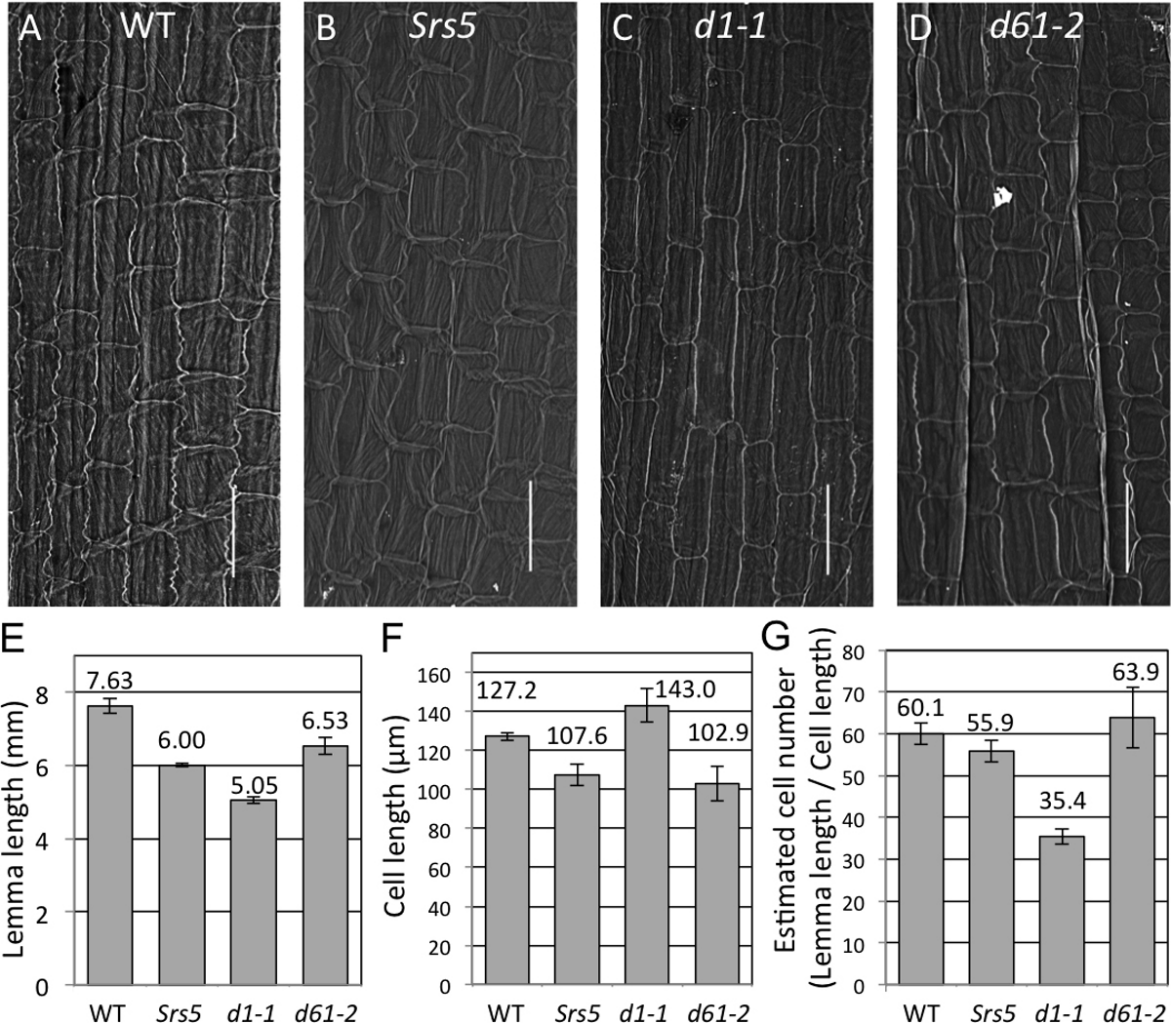
**Comparison of inner epidermal cell length of T65, *Srs5, d1-1*, and *d61-2***. Inner epidermal cells of the lemma of WT (A), *Srs5 *(B), *d1-1 *(C), and *d61-2 *(D) observed by SEM. Bars = 100 μm. (E) Lemma length of the WT, *Srs5*, *d1-1*, and *d61-2*. (F) Inner epidermal cell length of WT, *Srs5*, *d1-1*, and *d61-2*. (G) Estimated cell numbers of WT, *Srs5*, *d1-1*, and *d61-2*. Numbers in (E-G) indicate averages ± S.D.

To characterize short seed phenotype of *Srs5 *in detail, we compared the length of the inner epidermal cells of lemmas of *Srs5, d1-1*, and *d61-2 *using scanning electron microscopy (SEM). The cells of *Srs5 *were shorter than those of the WT (Figure 2A, B, and 2F), and similar to those of *d61-2 *(Figure 2D and 2F), but not those of *d1-1 *(Figure 2C and 2F). Additionally, we estimated cell numbers by dividing lemma length (Figure 2E) and by cell length (Figure 2F). Although *d1-1 *had a reduced number of inner epidermal cell of the lemma, the cell numbers of *Srs5 *and *d61-2 *were not significantly different from that of the WT (Figure 2G). From these observations, we concluded that the cause of the short seed phenotype of *Srs5 *is reduced cell length, as in *d61-2*.

### *SRS5 *gene encodes alpha-tubulin

**Figure 3:**
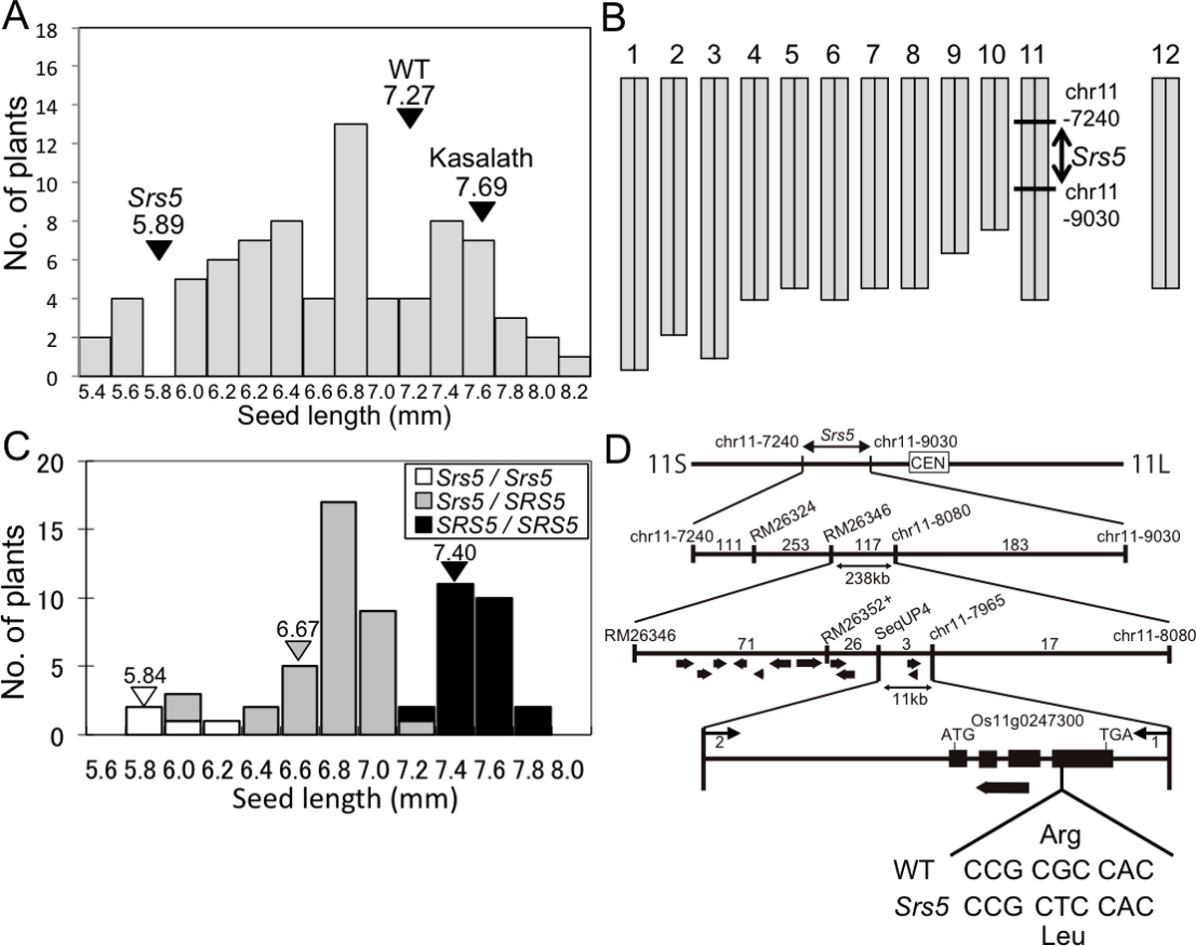
**Positional cloning of the *Srs5 *gene**. (A) Phenotypic distribution of F_3 _seed length obtained from F_2 _plants derived from a cross between *Srs5 *and Kasalath. Arrowheads indicate the average for WT, *Srs5*, and Kasalath. (B) *Srs5 *was mapped on the short arm of chromosome 11. (C) Phenotypic distribution of F_3 _seed length obtained from F_2 _plants derived from a cross between *Srs5 *and SL233. Arrowheads indicate the average for *Srs5/Srs5 *(white), *Srs5/SRS5 *(grey), and *SRS5/SRS5 *(black). (D) Linkage analysis of *Srs5*.

To map the *Srs5 *locus on rice chromosomes, we performed linkage analysis using F_2 _plants derived from a cross between the *Srs5 *mutant (*Oryza sativa*. ssp. *japonica*) and Kasalath (*Oryza sativa*. ssp. *indica*). Since F_2 _seeds show a continuous variation in seed length, short seed phenotypic expression of seed size seems to be affected by difference in genetic background between the *japonica *and *indica *subspecies, in addition to *Srs5 *locus (Figure 3A). From 2000 F_2 _plants, we obtained 13 F_2 _plants that produced evident short seeds in F_3 _progeny, indicating homozygous of *Srs5 *mutant gene. Linkage analysis revealed that *Srs5 *was located in the 1.8 Mb between chr11-7240 and chr11-9030 on chromosome 11 (Figure 3B). To further fine mapping of the gene, it was difficult to perform linkage analysis using this population, because of small number of plants with homozygous of mutant allele (small seed). For further analysis, we produced an F_2 _population derived from a cross between *Srs5 *and a Chromosome Segment Substitution Line (CSSL), SL233 that possessed the Kasalath chromosome segment of the short arm of chromosome 11 in a Koshihikari chromosome background. The phenotypes of F_3 _seeds obtained from these F_2 _plants were clearly distinguished mutant and wild types. From randomly selected 64 F_2 _plants, we obtained 24 WT plants (*SRS5/SRS5*), 36 heterozygote plants (*Srs5/SRS5*), and four mutant homozygote plants (*Srs5/Srs5*) (Figure 3C) with detecting the genotypes by using the PCR markers chr11-7240 and chr11-9030. The average seed lengths of *SRS5/SRS5*, *Srs5/SRS5*, and *Srs5/Srs5 *in the F_2 _population were 7.40 ± 0.15 mm, 6.67 ± 0.24 mm, and 5.84 ± 0.13 mm, respectively (Figure 3C). Since *Srs5/SRS5 *plants show seed lengths in-between those of *SRS5/SRS5 *and *Srs5/Srs5 *plants, the *Srs5 *mutant gene is considered to act as a semi-dominant manner (Figures 1A and 1D and 3C). The molecular markers chr11-7240 and chr11-9030 were used as selection markers for large-scale mapping (Figure 3B and 3D). We selected 664 from 5184 F_2 _recombinant plants that possessed chromosomal recombination within these two markers. We designed new molecular markers between chr11-7240 and chr11-9030, and 664 plants were used for high-resolution mapping (Figure 3D). The genotypes of F_2 _plant were confirmed by observation of the seed of F_3 _and F_4 _progeny. Finally, we obtained three recombinants within the 11 kb region including two predicted genes (RAP-DB http://rapdb.dna.affrc.go.jp/) (Figure 3D). Sequence analysis revealed only one single nucleotide polymorphism (SNP) in Os11g0247300 (Figure 3D). This mutation was resulted in an amino-acid substitution from Arg to Leu in residue 308 (Figure 3D).

### Complementation test

**Figure 4:**
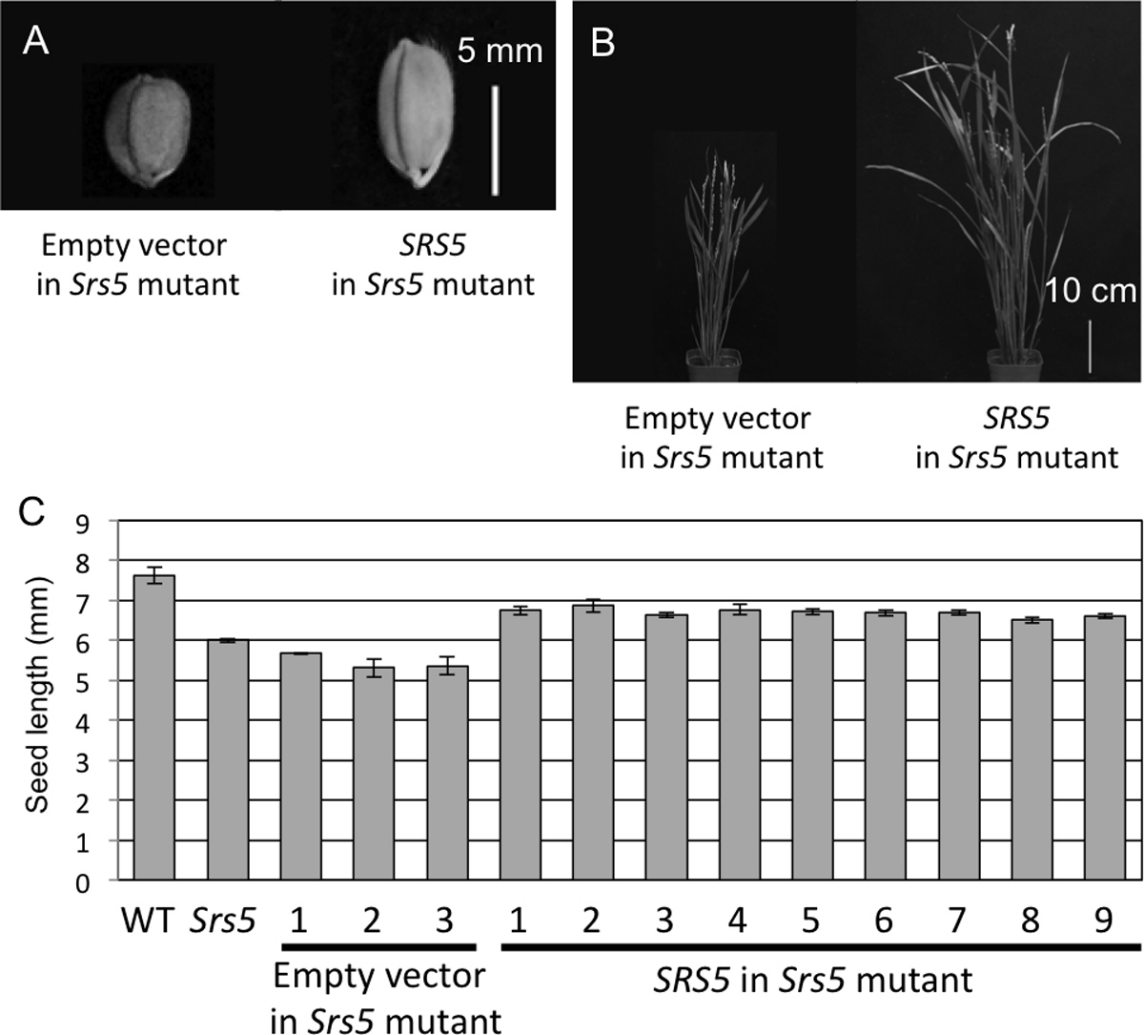
***Srs5 *complementation test**. (A) Seed morphology of transgenic plants. Bar = 5 mm. (B) Gross morphology of transgenic plants. Bar = 10 cm. (C) Seed length of transgenic plants. Numbers on graphs in (C) indicate averages ± S.D.

To confirm the SNP as causal mutation of *Srs5*, we performed a complementation test. The WT *SRS5 *gene was transformed into the *Srs5 *mutant. The seeds of positive transgenic plants were significantly longer than the seeds of those containing empty vector (Figure 4A and 4C). The dwarf phenotype was also rescued (Figure 4B), but phenotype of transgenic plant was not completely same as wild type.

### *SRS5 *expression

**Figure 5:**
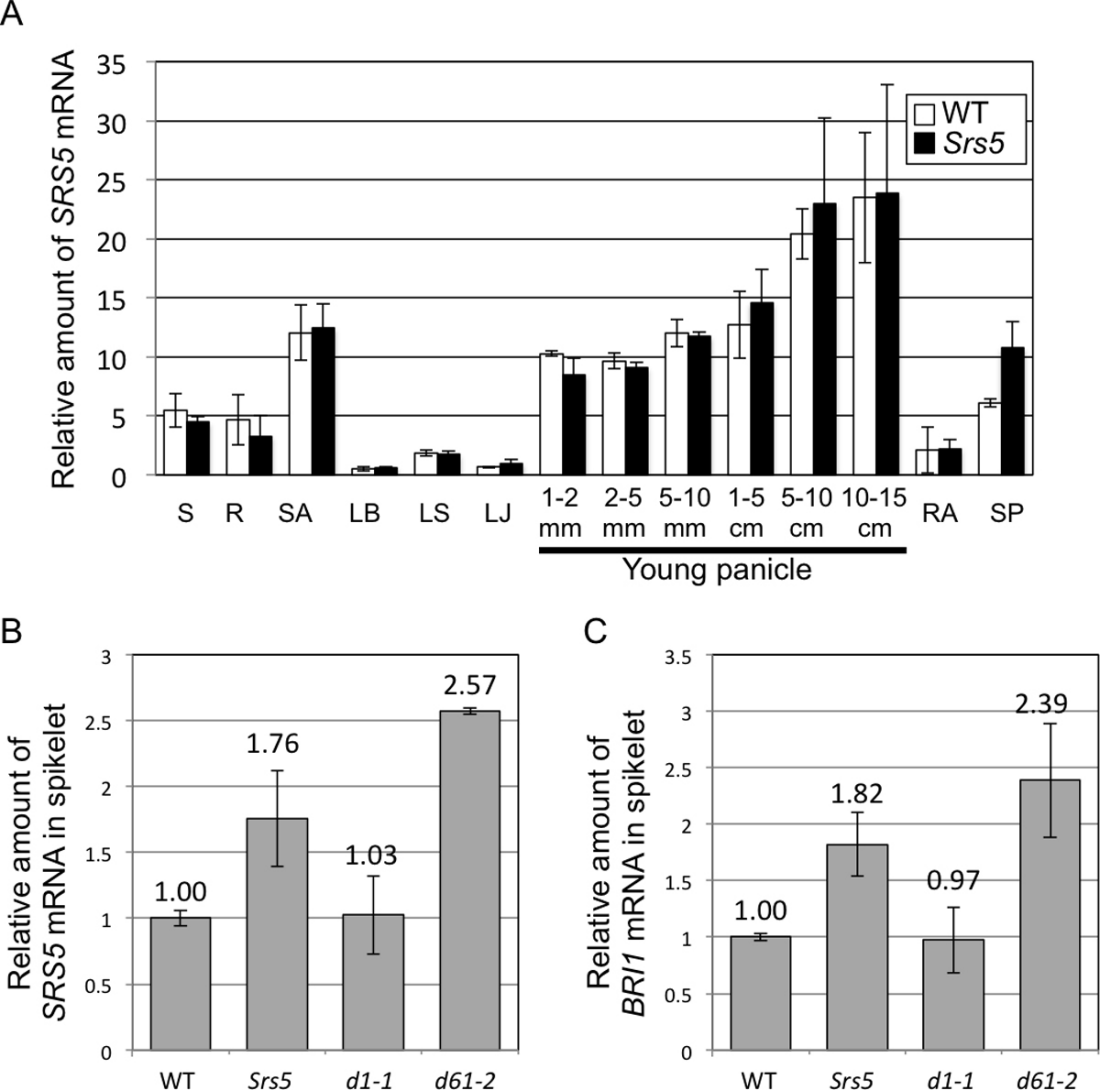
**Expression of *SRS5***. (A) Relative amounts of *SRS5 *mRNA in various organs were analyzed by quantitative RT-PCR. Abbreviations: S, Shoot; R, Root; SA, Shoot apex; LB, Leaf blade; LS, Leaf sheath; LJ, Lamina joint; RA, Rachis; SP, Spikelet. (B) Relative amounts of *SRS5 *mRNA in spikelet of WT, *Srs5*, *d1-1*, and *d61-2*. (C) Relative amounts of *BRI1 *mRNA in spikelet of WT, *Srs5*, *d1-1*, and *d61-2*. Bars represent S.D.

Accumulation of *SRS5 *mRNAs during the rice life cycle was investigated. *SRS5 *mRNA accumulated in the shoot apex, young panicles, and spikelets in both the WT and the *Srs5 *mutant (Figure 5A). In spikelets, accumulation of *SRS5 *mRNA was higher in the *Srs5 *mutant than in the WT (Figure 5A). Additionally, we compared the expression levels of *SRS5 *and *BRI1 *genes among *Srs5*, *d1-1*, and *d61-2 *mutants. Interestingly, we detected higher accumulation of *SRS5 *and *BRI1 *genes in both of *Srs5 *and *d61-2 *mutants (Figure 5B and 5C). In *d1-1 *mutant, the mRNA amounts of these genes were same level as WT (Figure 5B and 5C).

#### *Srs5 *regulates cell elongation independently of BR signal transduction

**Figure 6:**
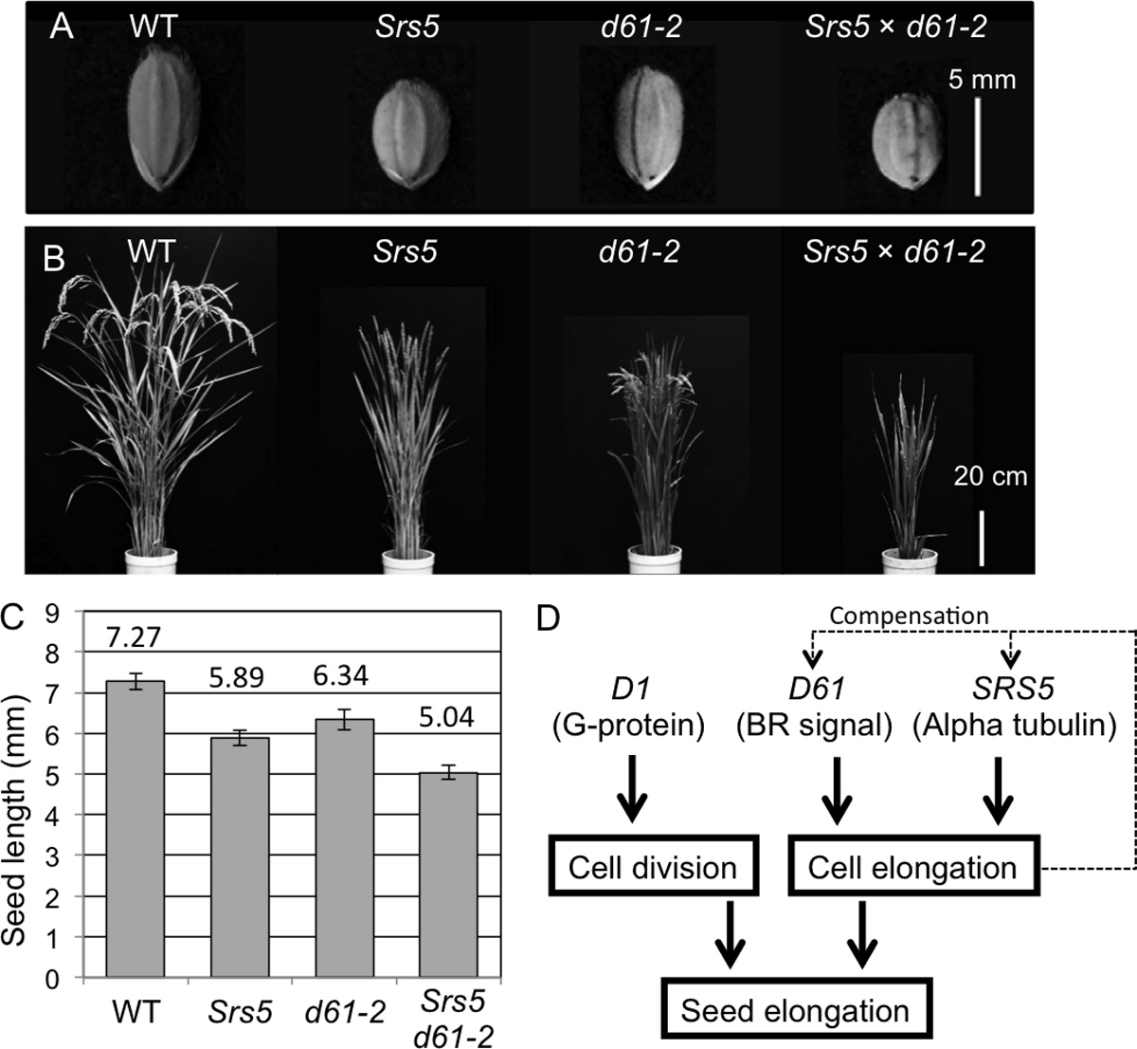
**Epistatic test of *Srs5 *and *d61-2***. (A) Seed morphology of T65, *Srs5*, *d61-2*, and *Srs5×d61-2*. Bar = 5 mm. (B) Gross morphology of T65, *Srs5*, *d61-2*, and *Srs5×d61-2*. Bar = 20 cm. (C) Seed length of T65, *Srs5*, *d61-2*, and *Srs5×d61-2*. Numbers indicate averages ± S.D. (D) Genetic model of *SRS5 *function during seed elongation in rice.

Since *Srs5 *exhibits shorter cells, as does *d61-2*, we produced a double mutant by crossing to determine whether these two genes have epistasis. The double mutant showed shorter seed length (Figure 6A and 6C) and plant height than both parent mutants (Figure 6B). This result indicates that *SRS5 *and *D61 *regulate cell elongation independently during seed formation.

## Discussion

In linkage analysis, we could not clearly distinguish seed size in F_2 _seed derived from distant cross between *Srs5 *and Kasalath. This was likely to be caused by background difference between indica and japonica. To overcome this, we used a chromosome segment substitution line (CSSL), which is a plant series that possesses relatively large chromosome segments of donor parent chromosomes in the recurrent parental chromosome background (Yano and Sasaki 1997, Yano 2001, Ebitani et al. 2005, Ashikari and Matsuoka 2006, Fukuoka et al. 2010). CSSLs can be used to achieve high accuracy in phenotyping in F_2 _populations. In fact, we could make classification of two seed size, wild and mutant type, in the F_2 _population derived from a cross between *Srs5 *and a CSSL.

Genetic analysis of the F_2 _population derived from a cross between *Srs5 *and SL233 demonstrated that the *Srs5 *gene act as semi-dominant manner. This semi-dominant effect was also confirmed in complementation test. Although The *Srs5 *mutants carrying WT *SRS5 *gene showed longer seeds than that of the plants containing empty vector, the degree of recovery was not completely same as WT (Figure 4B). The reason that the rescue by WT *SRS5 *gene was partial may be due to compete between WT and mutation gene products or incomplete conformation of the tubulin complex.

In this study, we demonstrated that the *SRS5 *gene encodes alpha-tubulin, which has been reported to be the causal gene of the rice mutation *Twisted dwarf 1*(*Tid1*) (Sunohara 2009). The *Tid1 *mutation acts as a semi-dominant gene by affecting the interaction of alpha and beta tubulin. Since *Srs5 *was also a semi-dominant mutation, it was likely caused by incomplete conformation of the tubulin complex. *Tid1 *shows right helical growth, in addition to a semi-dominant dwarf phenotype. Additionally, Arabidopsis *Lefty1 *and *Lefty2 *mutations in genes orthologous to *SRS5 *also show semi-dominant and left helical growth (Thitamadee *et al*. 2002). These two mutants were gain-of-function alleles and exhibited similar twisted plant phenotypes. As the *Srs5 *mutant does not exhibit a twisted phenotype, different mutations in alpha-tubulin seem to lead to different phenotypes. In spikelets, higher accumulation of *SRS5 *mRNA was detected in the *Srs5 *mutant than in the WT (Figure 5A). This seems to compensate for the reduced function of alpha-tubulin protein. Higher expression of *SRS5 *was also detected in *d61-2 *but not in *d1-1 *(Figure 5B and 5C). Furthermore, *BRI1 *gene highly expresses in *Srs5 *and *d61-2 *but not in *d1-1 *(Figure 5B and 5C). These results suggest that the expression of *SRS5 *and *BRI1 *genes are compensated by sensing the cell elongation inhibition in the *SRS5 *and *d61-2 *mutants, although *SRS5 *and *BRI1 *genes regulate cell elongation independently (Figure 6D). Three other alpha-tubulin genes are present in the rice genome, and they share a high homology (Sunohara *et al*. 2009). In organs that exhibited no significant change in phenotype in the *Srs5 *mutant, these alpha-tubulins might work redundantly to maintain rice body planning.

## Conclusions

Our study demonstrated that short seed mutants can be classified into two types: those with reduced cell numbers, e.g., *d1*, and those with reduced cell length, e.g., *d61 *(Figure 2A, C, and 2D). This facilitates classification of novel seed mutants. The short seed phenotype of *Srs5 *was demonstrated to be caused by reduced cell length, as in *d61*; however, the additive phenotype of the double mutant indicated that *SRS5 *and *D61 *regulate seed length via different mechanisms. To evaluate the mechanisms regulating seed length, observation of microtubule arrangement and analysis of double mutants among *srs1, srs3, Srs5*, and various BR mutants need to be performed.

## Methods

### Plant materials and growth conditions

Kyudai No. 37 was first identified at Kyushu University and maintained in Togo Field, Nagoya University. Its genetic background is unknown. A *japonica *cultivar, Taichung 65, was used as the WT plant. *d1-1 *was identified as spontaneous mutant 'Daikoku' and substituted its genetic background into Taichung 65 by backcrossing with Taichung 65 as a recurrent parent at Kyushu University. *d61-2 *was obtained by MNU treatment of Taichung 65. F_2 _and two parental lines were sown at the beginning of April. The seedlings of all plants were transplanted at the beginning of May into a paddy field at the Research Center for Bioresources Development in Fukui, Japan. They were then grown under natural conditions. Transgenic plants were grown in a closed greenhouse under natural sunlight. Room temperature was maintained at 30°C from 09:00 to 18:00 and 25°C from 18:00 to 09:00.

### Linkage analysis of *SRS5*

For mapping, F_2 _plants derived from a cross between *Srs5 *(*japonica*) and Kasalath (*indica*) were used. Genomic DNA was extracted from fresh leaf tissues of 13 F_2 _plants that exhibited the small and round seed phenotype by the CTAB method. The genetic linkage between the *Srs5 *locus and molecular markers was determined using the sequence tagged site (STS) and cleaved amplified polymorphic sequence (CAPS) markers reported by the Rice Genome Program and microsatellite markers (McCouch *et al*. 2002). F_2 _plants derived from a cross between *Srs5 *and SL233 were used for fine mapping of *Srs5 *gene. Recombinant plants possessing a recombination between PCR markers chr11-7240 and chr11-9030 were screened from 5184 F_2 _plants. Other markers on chromosome 11 were designed by comparing the sequences of *Srs5 *and SL233. Information on the PCR markers used in this study is shown in Table 1. Phenotypes were determined using F_3 _seeds obtained from F_2 _plants and F_4 _seeds obtained from F_3 _plants.

**Table 1:** Primer sequences used in this study

	Forward (5'→3')	Reverse (5'→3')
chr11-7240	GTTCATGTCCCTATCGATTC	GAGGACCTTATTGTTTGCC

RM26324	GAGATGGAGGGAGAAGCTACG	GTTCATTGGCATCATCAACC

RM26346	GCGCTTGTAGGAAGTTTAATGG	GTATCAGTGCTGGCTTGTAATACC

RM26352+	GCCTACCTTCAGCTTAAAACA	GTAAGATAAGTAAGACAACGAG

SeqUP4	GTTGTCTTTTCCAATTGTAGATA	CATACATAATATCCATAGACTATT

chr11-7965	GACGTTAACTAAGGCTGTGTT	GTTTAAGCTGTGTCTAGATCC

chr11-8080	CTCAGTTACTCTGATCTTCC	TCAAGCTTCTGTTCACAAGC

chr11-9030	TGCTCAGACCTTACAATGAG	TCAAACATGCACCAGAGTTC

alpha-tub-5kb-up	GTGCTCAAGATGGTCGATGA	GTGCTCAAGATGGTCGATGA

alpha-tub-intron	GCCATGATCCGTGCTGAAAT	GCCATGATCCGTGCTGAAAT

alpha-tub-exon	CGACAATGAGGCCATCTATG	CGACAATGAGGCCATCTATG

alpha-tub-5kb-down	GCTCTCTTCCAGAAATCAAGA	GCTCTCTTCCAGAAATCAAGA

RT-alpha-tub	ATGAGGGAGTGCATCTCGAT	CAAGATCGACGAAGACAGCA

RT-OsUbiquitin	CTTGGTCGTGTCCCGTTTC	TTCTTCCATGCTGCTCTACCAC

### Production of transgenic plants

The BAC clone containing the *SRS5 *gene was screened from the BAC library (constructed by the CUGI BAC/EST Resource Center) using four PCR primers, alpha-tub-5kb-up, alpha-tub-intron, alpha-tub-exon, and alpha-tub-5kb-down.

The BAC clone OSJNBa0014D2 was partially digested by *Sau*3AI and cloned into the *Bam*HI site of binary vector pYLTAC7 (Liu *et al*. 1999) (provided by RIKEN BioResource Center). This clone contains the 7.26 kb upstream region from the transcriptional start site of the *Srs5 *gene and the 1.13 kb downstream region from the end of the 3'UTR region of the *SRS5 *gene. The binary vector was transformed into *Agrobacterium tumefaciens *strain EHA105 (Hood 1993) by electroporation, and *Srs5 *mutants were transformed as reported previously (Ashikari *et al*. 2005). *Srs5 *mutants containing empty vectors were used as controls.

### RNA isolation and RT-PCR

Total RNA was extracted using the RNeasy Mini Kit (QIAGEN, Hilden, Germany). cDNAs were synthesized from total RNA using the SuperScript III system (Invitrogen, Carlsbad, CA, USA).

For quantification of *SRS5 *mRNA, real-time RT-PCR was carried out using SYBR Premix Ex Taq ™ II (TAKARA Bio, Inc., Tokyo, Japan). Two primers, RT-alpha-tub, were used to quantify *Srs5 *expression; a further two, RT-OsUbiquitin, were used to quantify *OsUbiquitin1 *expression (accession No. Os06g0681400). The Thermal Cycler Dice Real Time System (TAKARA Bio, Inc.) was used for quantification for real-time RT-PCR.

## Competing interests

The authors declare that they have no competing interests.

## Authors' contributions

SS carried out molecular genetic studies, expression analysis, electron microscopic analysis, and transgenic analysis, and wrote manuscript. IK carried out molecular genetic studies. TA carried out molecular genetic studies. MY carried out molecular genetic studies and wrote manuscript. HK provided all plant materials KM designed research, carried out molecular genetic studies, expression analysis, electron microscopic analysis, and transgenic analysis, and wrote manuscript. YI designed research and wrote manuscript. All authors read and approved the final manuscript.
